# Supplementary material for: QSAR Implementation for HIC Retention Time Prediction of mAbs Using Fab Structure: A Comparison between Structural Representations
Source: Int J Mol Sci. 2020 Oct 28;21(21):8037. doi: 10.3390/ijms21218037 (PMC7663183; doi:10.3390/ijms21218037)
Supplement: Supplementary file 1 [file ijms-21-08037-s001.pdf]

Supplementary Information

Supplemental Table 1 . Classification of mAb species using C-SVM on the Seq2D descriptor set. Model performance were assessed with Matthew's Correlation Coefficient (MCC), Error Rate (ER), Sensitivity (Sens) and Specificity (Spec) in calibration, cross-validation and external test set generated with the CADEX algorithm. Results for two models are shown, top values show performance of classification model based on the Jain dataset while the second is an extended dataset with 123 additional samples acquired from the IMGT database.

			Calibration				Cross Validation				Prediction			
**Data**	**Species**	**Samples**	**MCC**	**ER**	**Sens**	**Spec**	**MCC**	**ER**	**Sens**	**Spec**	**MCC**	**ER**	**Sens**	**Spec**
	Chimeric	10			1.00	1.00			0.29	0.99			1.00	0.93
Jain	Human	26	0.95	0.03	0.95	0.98	0.42	0.31	0.53	0.88	0.71	0.18	0.67	1.00
	Humanized	45			0.97	0.96			0.87	0.50			0.89	0.75
	Chimeric	30			0.96	1.00			0.62	0.97			0.83	1.00
Extended	Human	84	0.95	0.03	0.99	0.67	0.73	0.16	0.88	0.89	0.76	0.15	0.88	0.88
	Humanized	90			0.96	0.98			0.87	0.86			0.83	0.87

Supplementary Table S.2: List of generated descriptors from ProtDCal and EMBOSS Pepstats. The stars in the second and third columns represent which software was used for generation of each descriptor.

Descriptor	ProtDCal	Pepstats	Type	Description
$G_W(U)$	●		Thermodynamic	Energy contribution to the free energy from the entropy of the first shell of water molecules of a sequence in an unfolded state
$G_s(U)$	●		Thermodynamic	The interfacial free energy of a sequence in an unfolded state
$W(U)$	●		Thermodynamic	Empirical number of water molecules close to the sequence in an unfolded state
HP	●		Physiochemical	Hydrophobicity of sequence with Kyte-Doolittle scale
ECI	●		Physiochemical	Electronic Charge Index of sequence
ISA	●		Physiochemical	Isotropic Surface Area of sequence
A_p	●		Physiochemical	Polar area of sequence in an unfolded state
M_W		●	Physiochemical	Molecular weight of the sequence
IP		●	Physiochemical	Isoelectric point of the sequence
$Charge$		●	Physiochemical	The sum of all charges in sequence
AR_W		●	Physiochemical	Average residue weight
Res		●	Physiochemical	Number of residues in sequence

Supplementary Table S.3: Amino acid scales used for descriptor generation and details on captured information of the individual components

Scale	Description	Method	Number of Components	Component	Component descriptions
**Z-Scale**	Physiochemical	PCA	3	Z1	Contains information related to the hydrophobicity
				Z2	Contains information related to size, hydrophobicity and hydrophilicity
				Z3	Contains information related to pH and NMR values
**T-scale**	Topological	PCA	5	T1	No information given
				T2	No information given
				T3	No information given
				T4	No information given
				T5	No information given
**MSWHIM**	Electrostatic potential	PCA	3	MS1	Contains information related to the charge and size
				MS2	Contains information for further separation of positively charged residues
				MS3	Contains information for further separation of negatively charged residues

Supplementary Table S.4: List of energy and topological descriptors used to describe the protein structure

Descriptor	ProtDCal	GROMACS	Type	Description
$G_c(F)$	●		Thermodynamic	Contribution to the free energy from the conformational entropy in a folded state
$G_W(F)$	●		Thermodynamic	Contribution to the free energy from the entropy of the first shell of water molecules in a folded state
$G_s(F)$	●		Thermodynamic	Interfacial free energy of a folded state
$W(F)$	●		Thermodynamic	Number of water molecules close to a residue in a folded state
HBd	●		Thermodynamic	Number of hydrogen bond in the backbone of the protein
ΔG_s	●		Thermodynamic	Variation of the interfacial free energy between folded and unfolded states
ΔG_W	●		Thermodynamic	Contribution to the folding free energy of the first shell off water molecules
ΔG_{el}	●		Thermodynamic	Free energy contribution of the charge distribution within the protein
ΔG_{LJ}	●		Thermodynamic	Contribution of the Van der Waals interaction to the folding free energy
ΔG_{tors}	●		Thermodynamic	Contribution of the dihedral torsion potential to the folding free energy
$ln(FD)$	●		Topological	Logarithm of the folding degree
$SASA_{polar}$		●	Topological	The total solvent accessible surface area from the polar residues
SP_{polar}		●	Topological	The effective surface polarity from the charged and polar residues
$SASA_{non-polar}$		●	Topological	The total solvent accessible surface area from the non-polar residues
$SP_{non-polar}$		●	Topological	The effective surface hydrophobicity from the non-polar residues

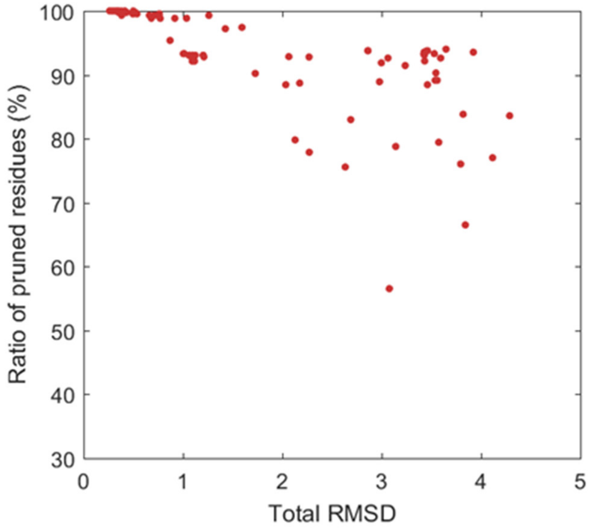

Supplementary Figure S.1: Superimposition of the 79 mAbs to the used template (PDB = 2FGW) with the UCSF Chimera MatchMaker function. The total RMSD for each superimposition is plotted against the ratio between pruned residues and the total number of residues in each mAb structure.

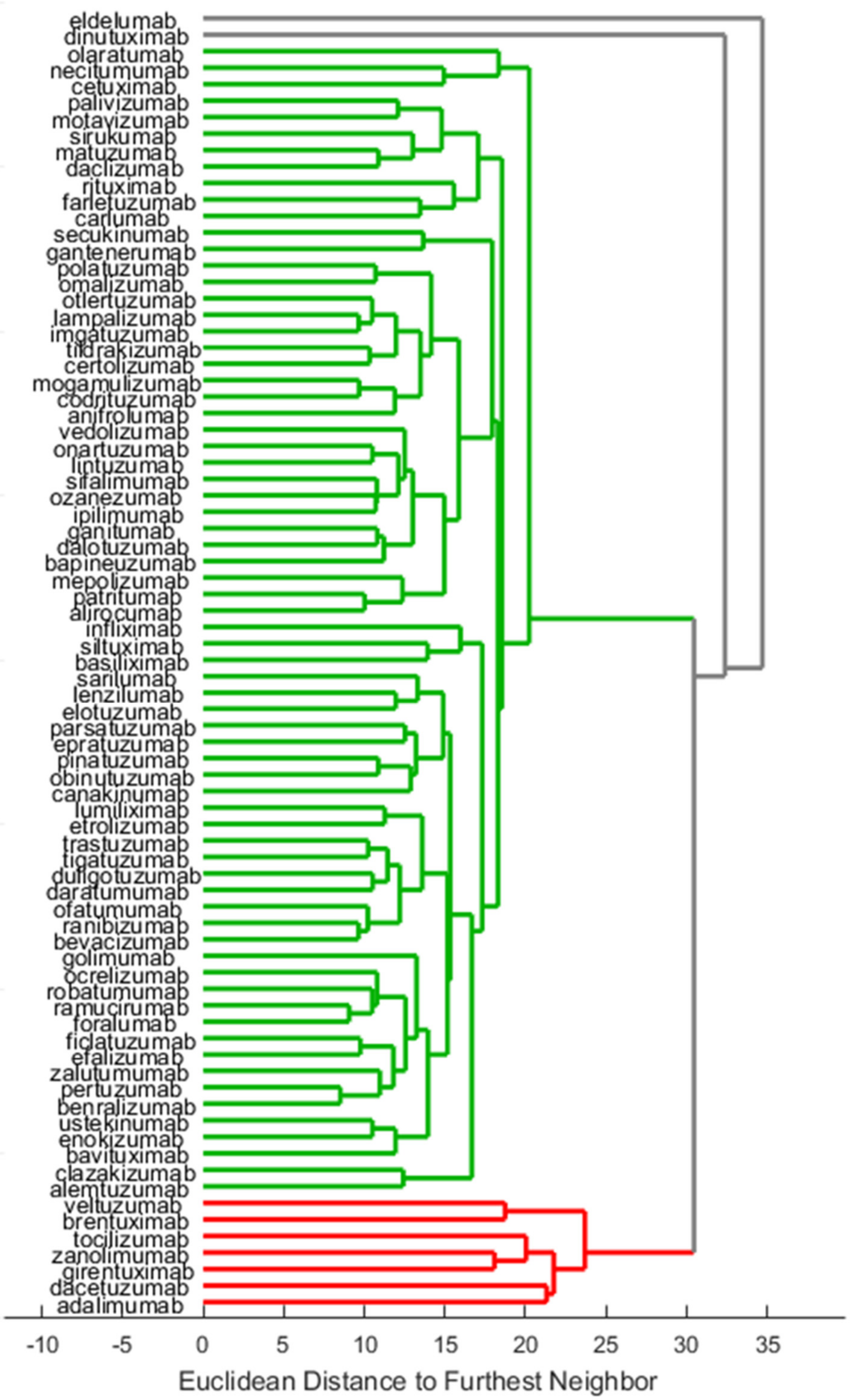

Supplementary Figure S.2: HCA dendrogram of the 79 mAb samples using the furthest neighbour algorithm and Euclidean distance in the MD3D descriptor space. Two clusters were observed shown in red and green whereas dinutuximab and eldelumab, shown in grey, were isolated from both clusters.

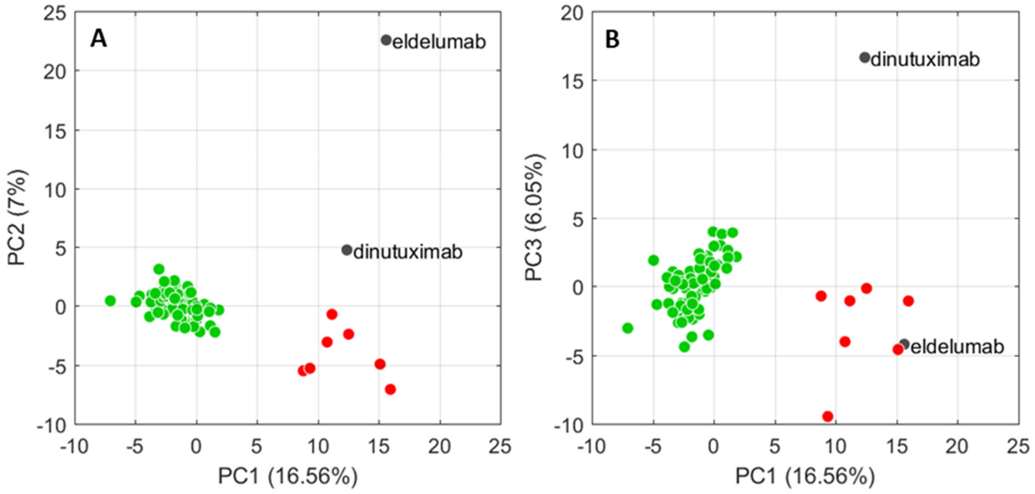

Supplementary Figure S.3: PCA scores of the 79 mAb samples obtained from the MD3D descriptors. The two clusters observed in HCA are depicted in the score plots in red and green. (**A**) Principal component 1 versus principal component 2. (**B**) Principal component 1 versus principal component 3.
